# Metabolomic Profiling of Plasma Reveals Differential Disease Severity Markers in COVID-19 Patients

**DOI:** 10.3389/fmicb.2022.844283

**Published:** 2022-04-27

**Authors:** Lucas Barbosa Oliveira, Victor Irungu Mwangi, Marco Aurélio Sartim, Jeany Delafiori, Geovana Manzan Sales, Arthur Noin de Oliveira, Estela Natacha Brandt Busanello, Fernando Fonseca de Almeida e Val, Mariana Simão Xavier, Fabio Trindade Costa, Djane Clarys Baía-da-Silva, Vanderson de Souza Sampaio, Marcus Vinicius Guimarães de Lacerda, Wuelton Marcelo Monteiro, Rodrigo Ramos Catharino, Gisely Cardoso de Melo

**Affiliations:** ^1^Programa de Pós-Graduação em Medicina Tropical (PPGMT), Universidade do Estado do Amazonas (UEA), Manaus, Brazil; ^2^Programas de Pós-Graduação em Imunologia Básica e Aplicada (PPGIBA), Universidade Federal do Amazonas (UFAM), Manaus, Brazil; ^3^Pró-reitoria de Pesquisa e Pós-graduação, Universidade Nilton Lins, Manaus, Brazil; ^4^Laboratório Innovare de Biomarcadores, Faculdade de Ciências Farmacêuticas, Universidade Estadual de Campinas (UNICAMP), Campinas, Brazil; ^5^Fundação de Medicina Tropical Heitor Vieira Dourado (FMT-HVD), Manaus, Brazil; ^6^Instituto Nacional de Infectologia Evandro Chagas, Fundação Oswaldo Cruz, Rio de Janeiro, Brazil; ^7^Instituto de Pesquisas Leônidas & Maria Deane (FIOCRUZ-Amazonas), Manaus, Brazil

**Keywords:** metabolomics, mass spectrometry, COVID-19, SARS-CoV-2, metabolites, prognosis

## Abstract

The severity, disabilities, and lethality caused by the coronavirus 2019 (COVID-19) disease have dumbfounded the entire world on an unprecedented scale. The multifactorial aspect of the infection has generated interest in understanding the clinical history of COVID-19, particularly the classification of severity and early prediction on prognosis. Metabolomics is a powerful tool for identifying metabolite signatures when profiling parasitic, metabolic, and microbial diseases. This study undertook a metabolomic approach to identify potential metabolic signatures to discriminate severe COVID-19 from non-severe COVID-19. The secondary aim was to determine whether the clinical and laboratory data from the severe and non-severe COVID-19 patients were compatible with the metabolomic findings. Metabolomic analysis of samples revealed that 43 metabolites from 9 classes indicated COVID-19 severity: 29 metabolites for non-severe and 14 metabolites for severe disease. The metabolites from porphyrin and purine pathways were significantly elevated in the severe disease group, suggesting that they could be potential prognostic biomarkers. Elevated levels of the cholesteryl ester CE (18:3) in non-severe patients matched the significantly different blood cholesterol components (total cholesterol and HDL, both *p* < 0.001) that were detected. Pathway analysis identified 8 metabolomic pathways associated with the 43 discriminating metabolites. Metabolomic pathway analysis revealed that COVID-19 affected glycerophospholipid and porphyrin metabolism but significantly affected the glycerophospholipid and linoleic acid metabolism pathways (*p* = 0.025 and *p* = 0.035, respectively). Our results indicate that these metabolomics-based markers could have prognostic and diagnostic potential when managing and understanding the evolution of COVID-19.

## Introduction

The new coronavirus 19 disease (COVID-19), which is caused by the severe acute respiratory syndrome coronavirus 2 (SARS-CoV-2) virus, was first reported in December 2019 in Wuhan, Hubei Province, China (Chan et al., 2020; Lauer et al., 2020). To date, more than 5 million people have died of the disease (WHO, 2021b). In most cases, infection with the SARS-CoV-2 virus is asymptomatic or clinically mild, but severe cases may occur and can lead to respiratory distress syndrome and even multiple organ failure (Monteiro et al., 2020; Zhu et al., 2020). Understanding the molecular events that underlie different clinical presentations and outcomes are urgently needed to help improve patient management (Pang et al., 2021).

Infectious diseases generate biological molecules that behave as markers for therapeutic and diagnosis/prognosis targets. Metabolomics analyzes the continuous communication between cells, tissues, and fluids through the exchange of metabolites in a given organism to give the precise status of the organism, organs, or tissues (Wishart et al., 2013; Dabaja et al., 2018; Chong et al., 2019; Li et al., 2020a). Thus, this branch of “-omics” is considered a hallmark approach in understanding disease pathophysiology. As such, the application of “-omics” sciences may play an essential role in the analysis of factors associated with gene function, transcription, mRNA degradation, post-translation modification, metabolite concentrations, flows, and other cell activity processes associated with disease (Goodacre et al., 2007; Borba et al., 2020).

The strong association among age, obesity, and diabetes and significant disease outcomes suggests that metabolic disturbances may play important roles in how the infection progresses (Haug et al., 2013). Recent studies have revealed critical metabolic dysregulations occurring in COVID-19 cases (Song et al., 2020; Wu et al., 2020a; Jimenez et al., 2021). In addition to mass spectrometry, other metabolomics techniques, such as nuclear magnetic resonance spectroscopy and liquid chromatography–mass spectrometry lipid profiling, have been employed (Kimhofer et al., 2020; Gray et al., 2021; Lorente et al., 2021; Meoni et al., 2021). In this context, an in-depth evaluation of metabolites, particularly those associated with different COVID-19 clinical presentations, is needed (Akarachantachote et al., 2014) and could further improve disease diagnosis, prognosis, or both, thus, potentially leading to personalized management strategies in the future (Blasco et al., 2020).

Due to the limited understanding of the biological mechanisms involved in a SARS-CoV-2 infection, the present study evaluated the metabolomic profile of the plasma of patients infected with SARS-CoV-2 in the Amazonas state, northern Brazil. The aim was to identify potential biomarkers of severity and to improve knowledge on the metabolic disturbances that take place following a SARS-CoV-2 infection, including the pathways associated with severe COVID-19.

## Methods

### Study Design and Patient Recruitment

Patients were recruited in Manaus, in the Western Brazilian Amazon, between March 2020 and June 2020 at the *Hospital e Pronto-Socorro Delphina Rinaldi Abdel Aziz*, which was the largest public hospital exclusively dedicated to treating severe COVID-19 in Manaus, Brazil, at the time.

The clinical trials recruited hospitalized individuals, as well as outpatients aged 18 years or older who were seeking care. In this study, patients who were hospitalized comprised the severe group, and these presented one or more of the following clinical symptoms: respiratory rate higher than 24 breaths per minute and/or heart rate higher than 125 beats per minute (in the absence of fever) and/or peripheral oxygen saturation lower than 90% in ambient air and/or shock (i.e., arterial pressure lower than 65 mmHg, with the need for vasopressor medicines, oliguria, or a lower level of consciousness in the last 7 days. Patients had to have had a confirmed laboratory diagnosis for COVID-19 *via* RT-PCR testing of nasopharyngeal swab sample (Huang et al., 2020; Qin et al., 2020; Zhou et al., 2020a).

Those seeking care, but who were not hospitalized (mild/moderate symptomatic SARS-CoV-2) and who tested positive for COVID-19 (RT-PCR) constituted the non-severe group for the current study. Individuals were considered to have mild illness if they presented various signs and symptoms of COVID-19 (such as fever, cough, sore throat, malaise, headache, muscle pain, nausea, diarrhea, and the loss of smell and taste), but did not have shortness of breath, dyspnea, or abnormal chest X-ray results. Patients displaying lower respiratory disease and having oxygen saturation above 94% in room air at sea level were considered to have moderate COVID-19 (National Institutes of Health, 2020a).

### Clinical Data Analysis

Clinical data and the patient's previous medical history were collected on admission (D0). Data included gender, age, weight, body mass index, presence of comorbidities such as hypertension, chronic cardiovascular diseases, chronic pulmonary diseases, current tuberculosis or history of tuberculosis (TB), HIV/AIDS, renal diseases, liver diseases, hematological conditions and diabetes, smoking history, and current use of medications or ongoing treatments. The collected data were managed using REDCap (v. 10.2.1) electronic data capture tools hosted at *Fundação de Medicina Tropical Dr. Heitor Vieira Dourado*, Manaus, Brazil.

### Laboratory Analysis

Hematological and biochemical analyses were automated. Complete blood count, platelets, creatinine, urea, ferritin, total cholesterol, low-density lipoprotein (LDL), high-density lipoprotein (HDL), lactate dehydrogenase (LDH), liver function tests [alanine transaminase (ALT), and aspartate transaminase (AST)], bilirubin (total, direct and indirect), C-reactive protein, IL-6, international normalized ratio (INR), creatine kinase (CK), creatine kinase myocardial band (CK-MB), troponin, sodium, and potassium analysis were performed.

### Metabolite Analysis

Samples of plasma metabolites from patients were extracted by mixing a 20 μL aliquot of each plasma sample with 200 μL of tetrahydrofuran and 780 μL of methanol. The mixture was homogenized using vortex agitation and centrifuged for 5 min, 3,400 rpm at 4°C. Subsequently, 5 μL of the supernatant was diluted in 495 μL of methanol. In this analysis, positively charged metabolites were obtained by adding 1 μL of formic acid.

All plasma samples were randomized and directly infused in a mass spectrometer (HESI-Q-Orbitrap, Thermo Scientific, Bremen, Germany) with a mass resolution of 140,000 FWHM. The mass spectrometer parameters were set as follows: positive mode, *m/z* range 200–1,700, 10 mass spectral acquisitions per sample, sheath gas flow rate 5 units, capillary temperature 320°C, aux gas heater temperature 33°C, spray voltage 3.70 kV, automatic gain control (AGC) at 1 × 10^6^, S-lens RF level 50, and injection time <2 ms. Full mass spectra were displayed using XCalibur 3.0 software (Thermo, Bremen, Germany).

Data were pre-processed and assessed for quality using an approach established in a previous study (Delafiori et al., 2021). In summary, as a quality control measure, the acquired replicates assisted us in discarding inconsistence during sample acquisition across batches and within patients' data. Noise peaks were filtered out when not present in at least 50% of scans in each acquisition. Additionally, the root-mean-square error (RMSE) was applied across technical replicates (Delafiori et al., 2021). Data were further processed by attributing an average for the 10 spectral acquisitions for each sample, followed by quantile normalization and logarithmic transformation. For the volcano plot, a fold-change (FC) threshold of 1.5 (severe/non-severe) and *p* < 0.05 were used as criteria for *m/z* feature selection using the MetaboAnalyst 4.0 online platform (Chong et al., 2019). Using the METLIN database (https://metlin.scripps.edu/landing_page.php?pgcontent=mainPage), metabolites were annotated with mass accuracy ≤ 5 ppm. Differential intensities of metabolites between severe and non-severe cases were evaluated by ranking log_2_FC scores and through heat map analysis/hierarchical clustering using the Ward clustering algorithm with Euclidean distance. To test the relevance of the selected metabolites in discriminating the groups, a partial least square-discriminant analysis (PLS-DA) score plot was projected and assessed using permutation tests (*p* < 0.01) (Blasco et al., 2020). Permuted data performance measures generally form normal distribution; the performance score of the original data outside the distribution qualifies the results as significant.

Databases, such as Human Metabolome Database (HMDB—www.hmdb.ca), LIPIDMAPS (www.lipidmaps.org), and Kyoto Gene and Genome Encyclopedia (KEGG—www.genome.jp/kegg), were used and supported bibliographic search bookmarks. Pathway analysis of annotated molecules with an available HMDB ID was evaluated against pre-established KEGG pathways for the human metabolism using MetaboAnalyst biomarker pathway analysis.

The potential of the annotated metabolites as biomarkers for predicting the severity of SARS-CoV-2 infection using the plasma metabolome profile of COVID-19 patients was explored and assessed via receiver operating characteristic (ROC) curves with confidence intervals (CIs) at a level of 95% using MetaboAnalyst 4.0. The ROC curve projected for each set of metabolites was an average plot of subsampling from 100 cross-validations. The confusion matrix generated by the selection of the linear-support vector machine algorithm for the classification of samples is computed as an average of predicted class probabilities of each sample across the 100 cross-validations. The algorithm uses a balanced sub-sampling approach, so the classification boundary is located at the center (*x* = 0.5). Additionally, data were validated by holding out 25% of samples as a test set, which lead to the evaluation metrics such as sensitivity, specificity, precision, and accuracy, and aimed to observe the differences in the performance of sample classification according to the set of metabolites selected. The process was repeated for 10 different sets of metabolites based on their relevance for PLS-DA variable importance in projecting (VIP) scores, log_2_FC, *p*, and area under the curve (AUC).

### Statistical Analysis

Comparison of continuous parameters that define the patient's characteristics, such as age, BMI, weight, the time between the onset of symptoms and hospitalization, and laboratory parameters were assessed using either the Student's *t*-test or Wilcoxon test, depending on the nature of data distribution (after the Shapiro–Wilk test for normality). Wilcoxon rank-sum test was used to analyze the differences in continuous variables with non-normal distribution between the two groups. Differences in categorical variables, such as gender, ethnicity, comorbidities, and medications, between the two patient groups were compared using the Chi-square test, with Fisher's exact test performed when variables were *n* < 5. Univariate logistic regression analysis was performed to study the associations between clinical variables and disease severity. Gender was used as a covariable in the multivariable analysis to for confounders.

Clinical and laboratory variables showing a strong association (*p* < 0.2) at the univariate level were included in the multivariate binary logistic regression to control for confounders while assessing the relationship between disease severity and clinical characteristics and/or laboratory parameters (Day 0). We chose *p* < 0.2 as an appropriate threshold for including variables in the multivariate model, as suggested elsewhere (Eskeziya et al., 2020). Statistical significance was established with a *p* < 0.05 and CIs at the 95% level. All statistical analyses were performed using Stata v.13 software (Stata Corp., TX, United States).

## Results

### Demographic Characteristics of the Patients

From the 242 recruited participants, 105 (43.4%) cases were identified as non-severe cases and 137 (56.6%) as severe cases (Table 1). Baseline characteristics showed a predominance of men 162 (66.9%), and the patients had a mean age of 51.0 ± 14.0. The majority of the patients identified themselves as being of mixed race (74.8%). A statistically significant association was observed between patient gender and disease severity (*p* < 0.001). Additionally, a significant difference was observed between males and females classified as severe, though no difference was observed in the non-severe group. The non-severe group had a mean age of 43.7 ± 12.4 years, compared to the severe group (56.6 ± 12.4 years). There was no significant difference between groups (*p* > 0.05) in terms of weight, BMI, and ethnicity. However, there was a significant difference in the time between onset of symptoms until hospitalization, with severe cases averaging 10.1 days, while non-severe cases averaging 8.8 days (*p* > 0.05).

**Table 1:** Demographics of patients at baseline.

**Variable**	**Total**	**Non-severe**	**Severe**	***p*-value**
	**(*N* = 242)**	**(*N* = 105)**	**(*N* = 137)**	
Sex, *n* (%)				<0.001
Male	162 (66.9)	50 (47.6)	112 (81.7)	
Female	80 (33.1)	55 (52.4)	25 (18.3)	
Ethnicity, *n* (%)				0.053
Mixed race	181 (74.8)	84 (80.0)	97 (70.8)	
European	39 (16.1)	14 (13.3)	25 (18.3)	
African	14 (5.8)	3 (2.9)	11 (8.0)	
Asiatic	5 (2.1)	4 (3.8)	1 (0.7)	
Amerindian	3 (1.2)	0	3 (2.2)	
Age, years				
Mean (SD)	51.0 (14.0)	43.7 (12.4)	56.6 (12.4)	<0.001
Weight, Kg				
Mean (SD)	81.2 (17.7)	80.7 (18.6)	81.6 (17.00)	0.5377
BMI, kg/m^2^				
Mean (SD)	29.4 (5.7)	29.6 (5.70)	29.3 (5.7)	0.4441
Onset of symptoms until admission, Days				
Mean (SD)	9.5 (5.8)	8.8 (6.0)	10.1 (5.6)	<0.001

Most of the patients (89.3%) reported having at least one comorbidity. A total of 85.7 and 92.0% of patients in the non-severe and severe groups, respectively, reported having a comorbidity (Table 2). A history of smoking (29.4%) and comorbidities such as hypertension (44.4%), and diabetes mellitus (33.3%), were most prevalent among the severe group patients, while obesity (50.0%), liver disease (10.0%), and chronic pulmonary disease (8.9%) were the comorbidities most prevalent among the non-severe group. At *p* = 0.005, the relationship between obesity and disease severity (non-severe or severe) was significant (Table 2). However, a proportion test revealed no significant difference in the proportions of obese patients in either of the two groups (*p* = 0.5139).

**Table 2:** Clinical and medical history of patients at baseline, with univariate and multivariate analysis of associations between COVID−19 severity and baseline characteristics.

**Variables**	**Number of patients, n/N (%)**			***p*-value**	**Univariate analysis**		**Multivariate analysis**	
	**Total**	**Non-severe**	**Severe**		**OR (95% CI)**	***p-*value**	**OR (95% CI)**	***p*-value**
	**(*N*, 242)**	**(*N*, 105)**	**(*N*, 137)**					
**Comorbidities**	**216/242 (89.3)**	**90/105 (85.7)**	**126/137 (92.0)**	**0.080**	**2.1 (0.902–4.887)**	**0.085**		
Chronic cardiac disease	14/216 (6.5)	5/90 (5.6)	9/126 (7.1)	0.855	1.2 (0.550–2.930)	0.584		
Hypertension	88/216 (40.7)	32/90 (35.6)	56/126 (44.4)	0.228	1.3 (0.750–2.250)	0.337		
Chronic pulmonary disease	18/216 (8.3)	8/90 (8.9)	10/126 (7.9)	0.233	0.6 (0.270–1.390)	0.245		
Previous tuberculosis	3/216 (1.4)	0	3/126 (2.4)	0.427	1.5 (0.620–3.550)	0.380		
Under treatment for tuberculosis	1/216 (0.5)	0	1/126 (0.8)	1.000	1.1 (0.458–2.778)	0.794		
Diabetes mellitus	66/216 (30.6)	24/90 (26.7)	42/126 (33.3)	0.301	1.2 (0.686–2.171)	0.498		
Obesity	84/216 (38.9)	45/90 (50.0)	39/126 (30.9)	0.005^*^	0.4 (0.256–0.785)	0.005	0.3 (0.092–0.875)	0.028
HIV/AIDS	6/216 (2.8)	3/90 (3.3)	3/126 (2.4)	0.213	0.6 (2.62–1.146)	0.110	0.3 (0.016–5.775)	0.425
Chronic renal disease	10/216 (4.6)	4/90 (4.4)	6/126 (4.8)	1.000	0.9 (0.415–2.041)	0.838		
Liver disease	14/216 (6.5)	9/90 (10.0)	5/126 (4.0)	0.191	0.6 (0.263–1.230)	0.151	0.6 (0.076–4.982)	0.650
Malignant neoplasm	1/216 (0.5)	0	1/126 (0.8)	1.000	0.9 (0.357–2.457)	0.901		
Chronic hematological disease	7/216 (3.2)	4/90 (4.4)	3/126 (2.4)	0.624	0.7 (0.312–1.684)	0.454		
Chronic neurological disease	10/216 (4.6)	2/90 (2.2)	8/126 (6.4)	0.392	0.6 (0.638–3.261)	0.379		
Rheumatic disorder	9/216 (4.2)	4/90 (4.4)	5/126 (4.0)	0.784	0.7 (0.289–1.700)	0.433		
Smoking:								
Former smoker	58/216 (26.9)	21/90 (23.3)	37/126 (29.4)	0.317	1.5 (0.818–2.872)	0.182	0.9 (0.266–3.242)	0.908
Current smoker	10/216 (4.6)	2/90 (2.2)	8/126 (6.3)		3.5 (0.714–16.961)	0.123		
Other relevant factors	8/216 (3.7)	1/90 (1.1)	7/126 (5.6)	0.228	1.4 (0.563–3.366)	0.484		
**Medications**	**219/242 (90.5)**	**88/105 (83.8)**	**131/137 (95.6)**	0.001*	**6.7 (2.227–20.404)**	**0.001**		
Ibuprofen	2/219 (0.9)	0	2/131 (1.5)	0.711	1			
Corticoids	26/219 (11.9)	7/88 (8.0)	19/131 (14.5)	0.228	2.1 (0.878–5.121)	0.095	1.6 (0.382–6.949)	0.510
Antibiotics:	148/219 (67.6)	32/88 (36.4)	116/131 (88.6)	<0.001^*^	13.5 (6.780–27.016)	<0.001		
Azithromycin	105/219 (47.9)	24/88 (27.3)	81/131 (61.8)	0.182	0.5 (0.175–1.409)	0.188	0.3 (0.086–1.154)	0.081
Other antibiotics	131/219 (59.8)	18/88 (20.5)	113/131 (86.3)	0.013^*^				
Bronchodilators	13/219 (5.9)	3/88 (3.4)	10/131 (7.6)	0.250	2.4 (0.631–8.837)	0.202	2.0 (0.287–13.774)	0.486
ACE Inhibitors	52/219 (23.7)	13/88 (14.8)	39/131 (29.8)	0.010^*^	2.4 (1.230–4.970)	0.011	0.9 (0.279–3.042)	0.893
Calcium blockers	7/219 (3.2)	3/88 (3.4)	4/131 (3.1)	1.000	1.3 (0.358–4.672)	0.694		
ARVs	4/219 (1.8)	3/88 (3.4)	1/131 (0.8)	0.305	0.2 (0.022–2.147)	0.193		
HCQ or CQ use in the last 30 days	7/219 (3.2)	0	7/131 (5.3)	0.004^*^	2.4 (1.21–4.76)	0.013		
Proton pump inhibitors	63/219 (28.8)	7/88 (8.0)	56/131 (42.7)	<0.001^*^	9.8 (4.233–22.687)	<0.001	3.1 (1.020–9.418)	0.046
Others	210/219 (95.9)	82/88 (93.2)	128/131 (97.7)	0.042^*^	4.7 (0.923–23.761)	0.062	8.9 (0.380–212.132)	0.173

* *Statistically significant. Bold values represents the total values for the total counts of Comorbidities and the Medications*.

Of the 242 participants, 219 (90.5%) were using medications at the time of hospitalization. Of whom, 83.8% (88) of the non-severe group, and 95.6% (131) of the severe group were already using assorted medications. The most commonly used drugs were antibiotics (67.6%), proton pump inhibitors (PPI, 28.8%), angiotensin-converting enzyme (ACE) inhibitors (23.7%), corticosteroids (11.9%), and other types of treatments (95.9%). Omeprazole and pantoprazole were the main PPIs. Patients in the severe COVID-19 group were the predominant users of antibiotics (88.6%), ACE inhibitors (29.8%), PPI (42.7%), and corticosteroids (14.5%). There was a significant difference in the two groups regarding the use of medications before hospitalization (p = 0.001). This difference was particularly observed concerning antibiotics in general (p ≤ 0.001), other non-azithromycin antibiotics (p = 0.013), ACE inhibitors (p = 0.010), hydroxychloroquine (p = 0.04), PPIs (p <0.001), and a variety of other medications (p = 0.041) (Table 2).

### Correlation of Disease Condition With Clinical and Laboratory Characteristics

#### Comorbidities and Use of Medications on Inclusion Into the Study

Overall, hypertension (40.7%), obesity (38.9%), and diabetes mellitus (30.6%) were observed to be the most prevalent comorbidities in the study participants. Univariate analysis showed no significant association between the presence of comorbidities and COVID-19 severity (p > 0.05) (Table 2). However, patients with comorbidities are likely to suffer severe COVID-19 (OR = 2.1, 95% CI = 0.900–4.880). The frequency of obesity was significantly higher in non-severe patients than in severe patients (p = 0.005). In addition, patients who used medications previously had a 6.7 times higher risk of developing severe ARDS (p = 0.001). Associations between drug use and severity were as follows: antibiotics (OR = 13.5, 95% CI = 6.780–27.016, p < 0.001), PPI (OR = 9.8, 95% CI = 4.233–22.687, p < 0.001), ACE inhibitors (OR = 2.4, 95% CI = 1.230–4.970, p = 0.011), HCQ/CQ (OR = 2.4, 95% CI = 1.21–4.76, p = 0.013), and bronchodilators (OR = 2.4, 95% CI = 0.631–8.837, p = 0.202) (Table 2).

Multivariate analysis suggested that obesity (OR = 0.3, 95% CI = 0.092–0.875, p = 0.028) was protective against severe disease, and patients with a history of using PPIs were significantly predisposed to developing a severe disease (OR = 3.1, 95% CI = 1.020–9.418, p = 0.046) (Table 2). The prior use of corticoids (OR = 1.6, 95% CI = 0.382–6.949, p = 0.510), bronchodilators (OR = 2.0, 95% CI = 0.287–13.774, p = 0.486), and other medications (OR = 8.9, 95% CI = 0.380–212.132, p = 0.173) predisposed patients to an increased risk of developing severe COVID-19, although not significantly.

#### Laboratory Parameters

As shown in Table 3, there were significantly higher levels of leukocytes, neutrophils, ALT, AST, direct and total bilirubin, HDL, creatinine, urea, LDH, and CKMB in severe compared to non-severe patients (*p* < 0.05). In contrast, lymphocyte, hematocrit, platelet, and total cholesterol levels were lower in patients in the severe group. Univariate logistic regression analysis showed that leukocyte counts (OR = 1.3, 95% CI = 1.198–1.446, *p* < 0.001), neutrophils (OR = 1.1, 95% CI = 1.088–1.159, *p* < 0.001), direct bilirubin (OR = 556.3, 95% CI = 36.277–8,530.587, *p* < 0.001), total bilirubin (OR = 7.2, 95% CI = 2.792–18.602, *p* < 0.001), and creatinine (OR = 2.9, 95% CI = 1.723–5.076, *p* < 0.001) were statistically significant laboratory indicators of severe illness. A univariate analysis further indicated that a decrease in lymphocyte count (OR = 0.8, 95% CI = 0.773–0.859, *p* < 0.001) and hematocrit (OR = 0.8, 95% CI = 0.812–0.916, *p* < 0.001) were negatively associated with disease severity (Table 3), thus suggesting that levels of lymphocyte count and hematocrit were inversely associated with disease severity.

**Table 3:** Baseline clinical laboratory characteristics of the enrolled subjects: comparison of laboratory parameters between groups and tests of association.

				**Wilcoxon rank-sum**	**Univariate analysis**
				**(Mann-Whitney) test**		
	**Total**	**Non-severe**	**Severe**	**p-value**	**OR (95%CI)**	**p-value**
	**(n)**	**median (IQR)**	**median (IQR)**			
**Parameters**
Leukocyte counts,103/mL	235	6.5 (5.3–8.8)	10.7 (7.8–13.7)	<0.001	1.3 (1.198–1.446)	<0.001
Lymphocyte counts, %	235	22.4 (15.2–31.3)	7.6 (3.9–11.6)	<0.001	0.8 (0.773–0.859)	<0.001
Neutrophil counts, %	235	68.7 (56.7–78.4)	87 (81.6–91.0)	<0.001	1.1 (1.088–1.159)	<0.001
Hematocrit, %	235	43.1 (40–46.1)	38.7 (35.3–41.9)	<0.001	0.8 (0.812–0.916)	<0.001
Platelet counts, 10^3^ /mL	235	248 (211–292)	221 (166.5–304.5)	0.030	1.0 (0.995–1.000)	0.099
INR	61	1.3 (1.1–1.4)	1.2 (1.1–1.3)	0.207	0.1 (0.000–8.914)	0.265
Alanine transaminase U/L	164	45.6 (29.0–66.5)	63.3 (38.1–86.8)	0.005	1.0 (1.000–1.015)	0.030
Aspartate transaminase U/L	163	36.5 (25.4–58.1)	63.6 (44.2–95.3)	<0.001	1.0 (1.016–1.041)	<0.001
Direct bilirubin, mg/dL	139	0.17 (0.12–0.23)	0.35 (0.2–0.77)	<0.001	556.3 (36.277–8530.587)	<0.001
Indirect bilirubin, mg/dL	139	0.19 (0.1–0.28)	0.21 (0.13–0.48)	0.112	4.3 (0.881–20.615)	0.072
Total bilirubin, mg/dL	139	0.34 (0.25–0.48)	0.66 (0.31–1.25)	<0.001	7.2 (2.792–18.602)	<0.001
Glucose, mg/dL	114	140 (127–301)	175 (132–242.5)	0.620	1.0 (0.994–1.009)	0.783
Total cholesterol, mg/dL	108	165.9 (137.7–197.4)	128.4 (109.7–153.6)	<0.001	1.0 (0.964–0.990)	0.001
HDL, mg/dL	83	43.4 (34.3–51.8)	27.1 (23.7–36.2)	<0.001	1.0 (0.999–1.008)	0.174
LDL, mg/dL	4	175 (n, 1)	93.83 ± 55.73 (n, 3)	ND		
Triglycerides, mg/dL	107	144.7 (103.6–274.6)	182.0 (137.1–236.4)	0.386	1.0 (0.996–1.002)	0.570
Creatinine, mg/dL	231	0.9 (0.7–1.1)	1.3 (0.9–2.5)	<0.001	2.9 (1.723–5.076)	<0.001
Urea, mg/dL	229	26.3 (21.9–30.7)	47.1 (31.1–87.9)	<0.001	1.0 (1.027–1.065)	<0.001
Lactate dehydrogenase U/L	81	665 (504–788)	997 (786–1,205)	<0.001	1.0 (1.002–1.007)	0.001
Creatine kinase U/L	174	94.5 (69.5–161.7)	11.1 (65.6–360.5)	0.126	1.0 (1.000–1.002)	0.020
Creatine kinase myocardial band U/L	140	19 (15.2–22.2)	22.9 (18.6–45.7)	<0.001	1.0 (1.010–1.065)	0.006
Alkaline phosphatase, U/L	7	84.1 (n, 1)	110.1 ± 47.38 (n, 6)	ND	1.0 (0.930–1.135)	0.598
Ferritin, ng/mL	76	ND	1,280 (843.5–1,950)	ND		
IL−6, pg/mL	70	ND	117,116 (75,772–228,959)	ND		
C-Reactive protein, mg/L	139	69.1 (38.5–79.3)	75.7 (67–85)	0.149	1.0 (0.996–1.031)	0.134
Sodium, mmol/L	140	139.5 (138.1–142.2)	140.6 (137.3–143.5)	0.423	1.0 (0.942–1.1522)	0.419
Potassium, mmol/L	139	4.1 (3.9–4.5)	4.3 (3.9–4.8)	0.367	1.4 (0.687–2.766)	0.367

*OR, odds ratio; IQR, interquartile range; SD, standard deviation; ND, not determined; INR, International normalized ratio; LDL, low-density lipoproteins; HDL, high-density lipoproteins; IL−6, interleukin 6*.

### Identification of Metabolites Related to SARS-CoV-2 Infection Severity and Pathway Analysis

An untargeted metabolomic approach was used to evaluate the metabolic profile of the COVID-19 patients. Data acquired in the positive ion mode within the mass range of 200–1,700 *m/z* were used to compare severe to non-severe cases *via* a volcano plot (log_2_Fc and *p*-value) with a fold-change threshold of 1.5 and *p* < 0.05. The volcano plot allowed us to display any large magnitude changes that are also statistically significant for ranking of *m/z* features (see Supplementary Figure 1). Selected m/z characteristics that had a large magnitude and were statistically significant subsequently underwent annotation using the metabolite databases resulting in a list of 43 annotated metabolites. Of the 43 metabolites, 29 were increased in the non-severe condition (but decreased in severe cases), while 14 metabolites were upregulated in the severe group (see Supplementary Table 1).

**Figure 1:**
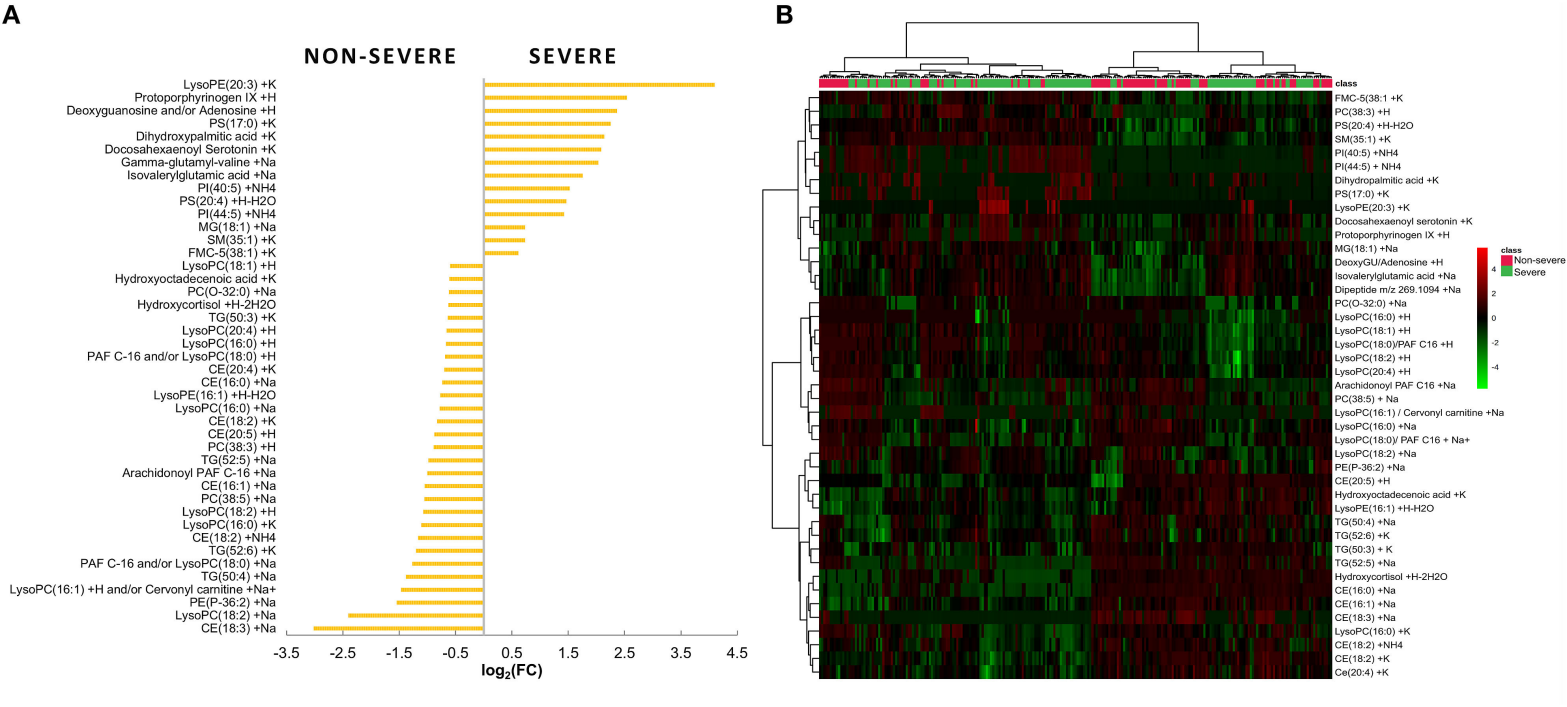
**(A)** Bar plot reporting the fold-change values of the 43 relevant metabolites selected using a combined evaluation of *p* and FC (severe/non-severe comparison). **(B)** Hierarchical clustering heat map showing the abundance of the top 43 metabolites based on VIP scores of non-severe and severe groups.

The annotated metabolites are shown in Figure 1. From the bar graph in Figure 1A, negative values of log_2_FC indicate that the metabolite was prevalent in the non-severe COVID-19 patients. In contrast, positive log_2_FC values were emblematic of a molecule's widespread distribution in the severe COVID-19 group. The heat map chart in Figure 1B illustrates the distribution of the metabolites across the two groups of patients based on the level of signal abundance. The bright red color indicates a higher/more intense signal abundance of a molecule within the groups, while a bright green color indicates a lower intensity of the metabolite.

Metabolites with log_2_FC > 2 or log_2_FC < −2 and *p* ≤ 0.001 were conspicuous: the cholesteryl ester CE (18:3), lysoPC (18:2), dipeptide, docosahexaenoyl serotonin, dihydroxypalmitic acid, PS (17:0), deoxyguanosine and/or adenosine, protoporphyrinogen IX, and lysophosphatidylethanolamine (lysoPE) (20:3). With the highest FC score (log_2_FC = 4.10), lysoPE (20:3) certainly stands out as a viable metabolite marker that is indicative of severe SARS-CoV-2 infection (Figure 1A). Metabolites CE (18:3) and lysoPC (18:2) were considerably abundant in the non-severe COVID-19 patients while deoxyguanosine and/or adenosine, protoporphyrinogen IX, and lysoPE (20:3) were conspicuously increased among the severe COVID-19 patients. Using VIP score (Akarachantachote et al., 2014), deoxyguanosine and/or adenosine was also identified as being a metabolite of interest and the main discriminating metabolite between the two groups of patients (see Supplementary Figure 2).

**Figure 2:**
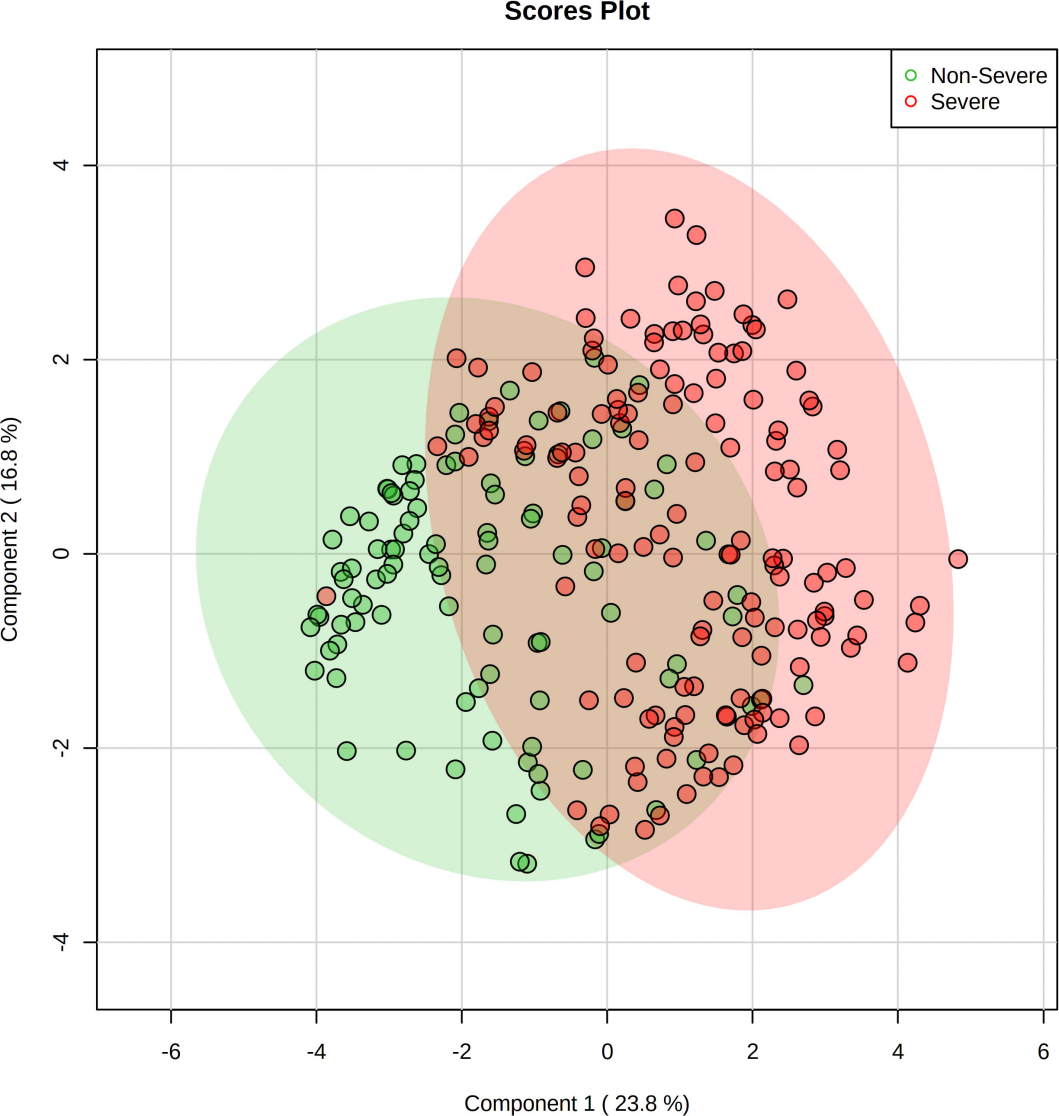
Supervised dimensionality reduction using partial least square-discriminant analysis (PLS-DA) showing the principal components (PC) score plot for non-severe (green) and severe (red) COVID-19 patients. Shaded areas show the 95% confidence regions, with 23.8% of variance explained by PC1 and 16.8% by PC2. Figure generated using MetaboAnalyst 4.0 (www.metaboanalyst.ca).

The annotated metabolites were used for multivariate statistical analysis using PLS-DA. The PLS-DA score plot indicated that the 43 selected metabolites were distinctly distributed between the two sets of patients, discriminating non-severe from severe groups (Figure 2) with an explained variation of 23.8% by PC1 and 16.8% by PC2. The model was validated using permutation tests, which were significant with a *p* < 0.001, thus indicating the robustness of the model (see Supplementary Figure 3).

**Figure 3:**
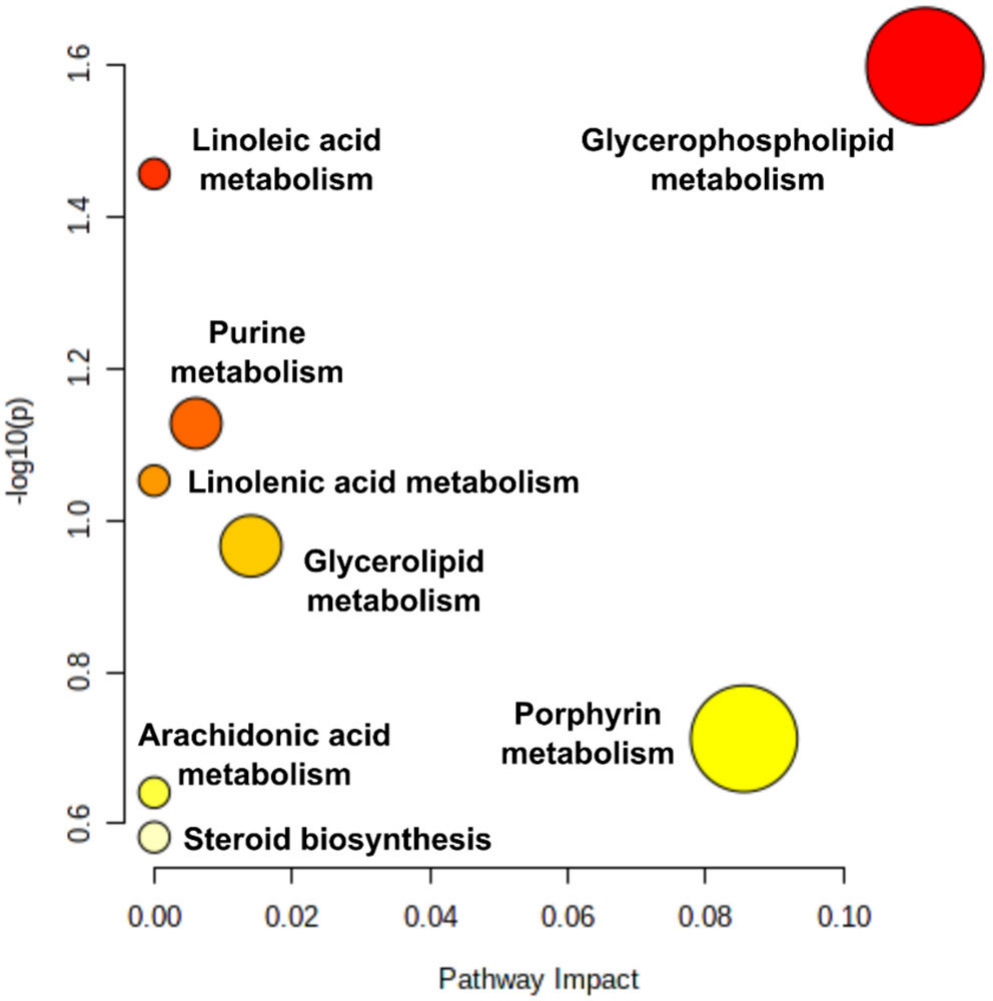
Pathway impact: pathway analysis based on enrichment analysis procedures, identifying the most relevant metabolic pathways *via* pathway impact and adjusted *p*. The figures were generated using MetaboAnalyst 4.0 (www.metaboanalyst.ca). Pathway impact here represents a combination of the centrality and pathway enrichment results; higher impact values represent the relative importance of the pathway; the size of the circle indicates the impact of the pathway while the color represents the significance (the more intense the red color, the lower the *p*).

Subsequent metabolic pathway analysis of the 43 annotated metabolites revealed that they were part of 8 metabolic pathways (Figure 3) glycerophospholipid, linoleic acid, purine, alpha-linolenic acid, glycerolipid, porphyrin and chlorophyll, arachidonic acid metabolism, and pathways of steroid biosynthesis. An analysis of the pathways identified that with higher log_2_FC impact values, two major pathways, namely, glycerophospholipid and porphyrin metabolism pathways, had the largest and most important changes within the conditions of patients with COVID-19 (Figure 3). Interestingly, glycerophospholipids were the main metabolite class predominantly associated with non-severe cases, with 16 out of the 21 representative phospholipid metabolites (see Supplementary Table 1). A large change was detected between the two groups in the porphyrin metabolism pathway. Overall, the glycerophospholipid and linoleic acid pathways had significant changes in metabolism between the two groups to assess the severity of COVID-19 (*p* = 0.025 and *p* = 0.035, respectively). Although the porphyrin metabolism pathway showed a high impact, it was not significant when compared to the glycerophospholipid metabolism pathway (*p* > 0.05).

#### Using the Metabolomic Profile to Discriminate Non-severe From Severe SARS-CoV-2 Infections

Receiver operating characteristic curves were obtained based on different sets of metabolites to determine the suitability of annotated markers for assessing the disease severity profile and ascertain metabolite placement prediction in the recruited study cohort. Sensitivity, specificity, precision, and accuracy values were used to evaluate the independent performance predictions using 25% holdout samples. As metabolite selection criteria, we used either single metabolites or a combination of ranked metabolites according to AUC, VIP score, *p*, and log_2_FC scores and group (see Supplementary Table 1) as summarized in Table 4.

**Table 4:** Summary of receiver operating characteristics (ROC) curve metrics discrimination of non-severe and severe COVID−19 disease condition based on the metabolic profile.

		**AUC**		**VIP score**		**Severe**		**Non-severe**		**Non-severe**
										**and severe**
**Performance**	**All**	**Top 5**	**Top 10**	**>1.5**	**≥1.0**	***p*-value**	***p*-value ≤0.01**	***p*-value**	***p*-value ≤0.01**	***p*-value ≤0.01**
	**markers**					**<0.01**	**log_**2**_(FC) > |2.0|**	**<0.01**	**log_**2**_(FC) > |2.0|**	**log_**2**_(FC) > |2.0|**
Metabolites (n)	43	5	10	5	15	10	7	18	2	9
AUC-ROC (100 CV)	0.877	0.860	0.882	0.866	0.865	0.861	0.859	0.883	0.818	0.865
CI 95% (100 CV)	0.827–0.946	0.805–0.908	0.824–0.932	0.801–0.929	0.803–0.921	0.801–0.926	0.794–0.918	0.824–0.939	0.738–0.897	0.791–0.927
AUC-ROC (25% holdout sample)	0.894	0.889	0.899	0.875	0.853	0.875	0.850	0.919	0.792	0.870
Sensitivity (%)	86.11	80.56	83.33	83.33	88.89	83.33	80.56	91.67	94.44	88.89
Specificity (%)	76.92	73.08	80.77	76.92	76.92	80.77	76.92	73.08	57.69	69.23
Accuracy (%)	81.52	76.82	82.05	80.13	82.91	82.05	78.74	82.37	76.07	79.06
Precision (%)	83.78	80.56	85.71	83.33	84.21	85.71	82.86	82.50	75.56	80.00

*FC, fold change; AUC, area under curve; SEN, sensitivity; SPE, specificity; PRE, precision; ACC, accuracy; CI, confidence interval; Best score in each ROC metric (sensitivity, specificity, precision and accuracy)*.

*The metrics were obtained using different sets of metabolite biomarkers*.

**Figure 4:**
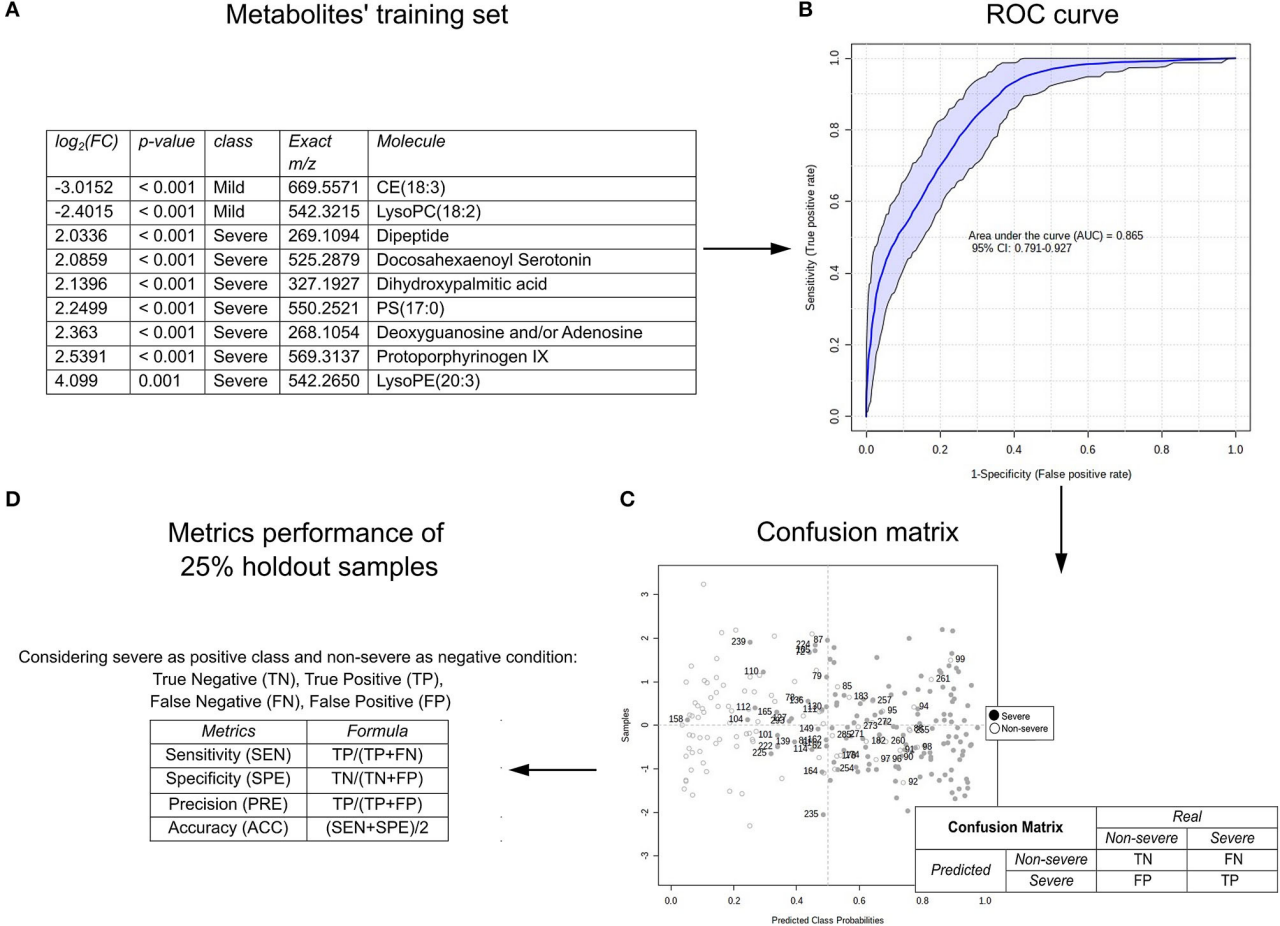
An example of the prediction of SARS-CoV-2 infection severity according to the metabolomic profile of the plasma of the COVID-19 patients using the receiver operating characteristic (ROC) curve; **(A)** set of metabolites used for training; **(B)** ROC curve evidenced by the area under the curve (AUC) and CI 95%; **(C)** confusion matrix for calculating the proportion of false negatives, false positives, true negatives, and true positives; **(D)** performance metrics as sensitivity, specificity, accuracy, and precision. The figures were generated *via* MetaboAnalyst software v 4.0 (www.metaboanalyst.ca).

Multiple ROC curves were obtained using either a single or a combination of the AUC marker characteristics, VIP-score, *p*, and log_2_FC for the ROC curve models. Figure 4 represents the analysis performed for the set of metabolites classified by log_2_(HR) > 2.0 and *p* ≤ 0.01 for the severe and non-severe groups. In summary, the process comprises the selection of a set of metabolites (Figure 4A) used for training the ROC curve (Figure 4B) where the AUC and the 95% CI for the model are given as a result of the correct classification of the patient in the confusion matrix (Figure 4C), which leads to the possibility of calculating performance metrics (Figure 4D). In the case of Figure 4, nine metabolites (considering the increased and decreased metabolites during severe COVID-19) provided the best discrimination performance with an AUC of 0.865 (95% CI: 0.791–0.927) and performance metrics with a sensitivity of 88.89, 69.23% specificity, 80.00% accuracy, and 79.06% accuracy for both COVID-19 disease severity categories and metrics. Comparing the 10 ROC curves designed using sets of metabolites, the assigned AUC–ROC ranged from 0.818 to 0.883, considering 100 cross-validations. The summary of AUC–ROC, CI 95% values, and performance metrics obtained are shown in Table 4.

Overall performance of all sets tested with 25% holdout samples was good at discriminating the two conditions with both a minimum of 5 metabolites and a maximum of 43 (Table 4). Using only 5 metabolites gives lower sensitivity, specificity, accuracy, and precision than using 10 or all the metabolites. VIP scores of ≥1.0 (15 metabolites) offered slightly better specificity, precision, and accuracy than > 1.5 (5 metabolites) (88.89 *vs*. 83.33, 84.21 *vs*. 83.33%, and 82.91 *vs*. 80.13%, respectively). Overall, it was observed that the best score for each of the ROC curve metrics concerning sensitivity, specificity, precision, and accuracy metrics were 94.44, 80.77, 85.71, and 82.91%, respectively (demarcated in Table 4), which were obtained under different selection criteria. When selecting metabolites that are indicative of severe and non-severe cases together, the best parameters were molecules with a *p* < 0.01, which are the same parameters chosen when selecting only non-severe COVID-19 markers; at a *p* < 0.01, strong metrics for model performance are maintained. However, this comes at the cost of not knowing the up-or-down-regulation levels of metabolites (Log_2_FC). Log_2_FC is vital in identifying the order of magnitude and importance of the most decreased or increased metabolites, based on the lowest or highest log_2_FC, respectively. Thus, having prediction criteria that comprised both severe and non-severe COVID-19 metabolites, such as those with the top 10 AUC results, may result in an equilibrated and good performance and are suitable for further investigation of the disease prognosis (Table 4).

## Discussion

COVID-19 is a multifactorial disease that results in asymptomatic infection, mild and moderate disease, and severe and fatal outcomes. Worldwide, its severity has already been associated with age, comorbidities, and other clinical or demographic conditions (Huang et al., 2020; Costa et al., 2021). Two years after the WHO declared COVID-19 a pandemic, giving a timely prognosis is still a complex challenge. The capacity to quickly and accurately identify factors associated with COVID-19 severity is of most importance.

In a systematic review and meta-analysis, Gold et al. (2020) identified the comorbidities that were most often associated with COVID-19. The findings provided an opportunity to further research the interaction between the underlying diseases and the pathophysiology of SARS-CoV-2 infection. Subsequently, to advance research on COVID-19 mechanisms, we aimed to understand the pathophysiology of the disease by interconnecting several factors, primarily through analyzing the metabolomic profile involved in COVID-19 severity Our findings corroborate results from other studies in which factors such as underlying morbidities like hypertension and diabetes were more prevalent in patients with severe COVID-19, with a tendency to progress to severe disease and an increased risk of death (Dai et al., 2020; Gold et al., 2020; Grasselli et al., 2020; Li et al., 2020b).

Although obesity exacerbates the risk of COVID-19 severity, the multivariate analysis results herein suggested that obesity had a protective role against disease severity. However, the perceived protective effect could be because of the small sample size used in the univariate and multivariate analysis, and not obesity itself. On the contrary, from the average BMI of the patients in the study (Table 1), of which all of them suffered varied severities of COVID-19, all were classified as being overweight or obese (WHO, 1995). From our analyses, it was possible to deduce that obesity was a crucial underlying factor, as demonstrated by other studies which considered obesity to be a predictor of COVID-19 disease severity (Yu et al., 2021; Zhou et al., 2021). With COVID-19, obese patients are more susceptible to infections and often require oxygen support when hospitalized (Liu et al., 2020a; Qiu et al., 2020; Ryan et al., 2020).

Despite the high frequency of chronic lung disease (8.9%) among the non-severe cohort of patients, there was no significant difference in chronic lung diseases between the two groups. Nonetheless, the absence of a statistically significant difference could be attributed to the small sample size. However, other studies have established that chronic lung disease is directly associated with lung injury by COVID-19 (Jimenez et al., 2021). While upregulation of ACE-2 is protective against severe lung injury, the subsequent high expression of ACE-2 receptors predisposes lung injury patients to an increased risk of SARS-CoV-2 infection of which the SARS-CoV-2 virus uses the ACE receptors to enter epithelial cells that line the lungs (Jimenez et al., 2021).

Since the declaration that COVID-19 was a pandemic, numerous studies have conducted therapeutic trials using unsanctioned WHO or Food and Drug Administration (FDA) treatments in the search for a cure for COVID-19 (Chen et al., 2020; Colson et al., 2020; Ghosh et al., 2020; Leung et al., 2020; Rochwerg et al., 2020). From our analysis, patients previously on treatment or using medications for other conditions were up to seven times more likely to develop severe COVID-19. Our study showed that the use of antibiotics, ACE inhibitors, PPIs, or HCQ/CQ at the time of hospital admission due to SARS-CoV-2 infection was more significantly associated with severe symptoms of COVID-19. In some studies, however, patients on ACE inhibitors and azithromycin were not at increased risk of poor outcomes from COVID-19 (Butler et al., 2021; Lopes et al., 2021; Pettit et al., 2021). Nevertheless, other studies have shown higher risks of deaths or side effects associated with complications related to the use of PPIs and HCQ/CQ (Cao et al., 2020; Ejaz et al., 2020; Ghazy et al., 2020; Jimenez et al., 2021). From our cohort, patients using corticoids at the time of recruitment were twice as likely to experience a severe COVID-19 infection. Although the use of corticosteroids was not helpful against the SARS and MERS-CoV diseases (Varghese et al., 2020; Cui et al., 2021), corticosteroids still are recommended as a treatment for only moderate, severe, and critical SARS-CoV-2 infection (National Institutes of Health, 2020b; Cui et al., 2021; Patel et al., 2021; Ro et al., 2021). The dose used and duration of treatment with corticosteroids remains contentious.

Analysis of laboratory parameters revealed an increase in neutrophil count, LDH, IL-6, AST, ALT, direct and total bilirubin, leukocyte count, creatinine, urea, ferritin, and CKMBM levels in severe cases. Based on recent studies, patients classified as having severe infection had elevated levels of LDH, IL-6, and leukocytes (Wang et al., 2020; Wu et al., 2020b; Zhou et al., 2020b). Progressive changes in these hematological and inflammation parameters, particularly LDH, IL-6, and white blood cell count, can serve as prognostics for severe disease capable of evolving to critical or fatal outcomes. Our data show that IL-6 levels were increased in critically ill patients, thus corroborating studies demonstrating that IL-6 gradually increased during hospitalization due to increased inflammation and worse evolution (Grifoni et al., 2020; Wang et al., 2020; Zhou et al., 2020b). Our findings support observations that increased IL-6 levels in patients with COVID-19 may be a predictor of progression of COVID-19 infection (Witt et al., 2018; Tang et al., 2020). These findings were followed by an elevation of neutrophils, significantly associated with the risk of developing severe ARDS in patients with COVID-19, which demonstrates an evident inflammatory response due to viral infection (Narasaraju et al., 2011; Cavalcanti et al., 2020; Fan et al., 2020; Huang et al., 2020; Mahévas et al., 2020; Nusbaum, 2020; Pagano et al., 2020; Sanders et al., 2020; Sharma et al., 2020; Struwe et al., 2020; Terpos et al., 2020; Young et al., 2020).

Metabolomic analysis showed a distinct profile of molecules in patients with severe COVID-19, with an increased presence of phospholipids, glycerolipids, *N*-acyl serotonin, porphyrin, purine, sphingolipids, sterols, unsaturated fatty acids, and amino acids. These observations corroborate the metabolic characterization study on COVID-19 patients by Shen et al. (2020). From the fold-change analysis of severe COVID-19 metabolites, the positive/higher the log_2_FC value the more the metabolite is detected, and the negative/lower the log_2_FC value the scarcer the metabolite in the severe case (or the more the metabolite is elevated in the non-severe COVID-19 situation). The severe COVID-19 patients expressed significantly high levels of protoporphyrinogen IX (porphyrin) and deoxyguanosine and/or adenosine (purine). Protoporphyrinogen IX and deoxyguanosine (or adenosine) could potentially serve as biomarkers for COVID-19 prognosis. The elevated porphyrin levels in the COVID-19 patients corroborated observations by Bruzzone et al. (2020), in which elevated porphyrin levels in serum were synonymous with porphyria arising from thrombocytopenia that implied liver damage, a key observation in our cohort of severe COVID-19 patients. Interestingly, lysoPE (20:3) also had the highest FC but had lower VIP scores than protoporphyrinogen and deoxyguanosine (and/or adenosine).

The metabolomic analysis of the plasma samples revealed elevated expression of deoxyguanosine (dG) in the severe group. This observation matched findings from other studies which associated the metabolite with an inflammatory immune reaction (Davenne et al., 2020; Delafiori et al., 2021). Since it was impossible to separate dG and the adenosine detected according to the *m/z* and the methods used, here, it is plausible that the relative quantity of either metabolite may have been an influencing factor. Deoxyguanosine (dG) triggers cytokine production in murine bone marrow–derived macrophages, plasmacytoid dendritic cells, as well as in human peripheral blood mononuclear cells, type I interferons, and pro-inflammatory factors, such as TNF and IL-6 (Davenne et al., 2020). A “cytokine storm” response to a SARS-CoV-2 virus infection characterizes severe COVID-19 disease, and excessive recruitment of macrophages from the peripheral blood results in acute lung injury (Delafiori et al., 2021). The elevated dG levels in this group of patients likely triggered the “cytokine storm” by inducing the production of IL-6 through the increased leukocyte population and the resulting inflammatory response. The overproduction of IL-6 mediates other reactions such as the elevated expression of adhesion molecules and cytokines (e.g., IL-1β and TNF-α) in endothelial cells that potentially increase the inflammatory response (Sprague and Khalil, 2009). This increased inflammatory response may be the direct role played by IL-6, which is the metabolite that is predominantly expressed in the pathology of the SARS-CoV-2 immune response, in addition to being an endogenous biomarker of oxidative stress. Oxidative stress and pro-inflammatory cascades are strongly related. Nevertheless, it remains unclear what triggers increased dG production during SARS-CoV-2 infection (Shen et al., 2020).

Pathway analysis approaches use available pathway databases and the given gene expression data to identify the pathways that are significantly impacted in a given condition (Nguyen et al., 2019). Pathway analysis revealed that the 43 differentially expressed metabolites were from 8 main metabolic pathways: glycerophospholipid, linoleic acid, purine, alpha-linolenic acid, glycerolipid, porphyrin and chlorophyll, arachidonic acid metabolism, and biosynthesis pathways of steroids (Figure 3). The two primary metabolites, lysoPE (20:3) and protoporphyrinogen IX, suggested that the glycerophospholipid and the porphyrin metabolic pathways have a role in the progression of clinical disease. Further analysis demonstrated that activity in the glycerophospholipid and linoleic acid pathways significantly differed across the two COVID-19 clinical presentations (Figure 3). The elevated expression of phosphatidylcholines and lysophosphatidylcholines in the non-severe group implied suppressed glycerophospholipid and linoleic acid pathway activities in severe COVID-19 patients. It is also highlighted that the porphyrin metabolism pathway in the severe COVID-19 group was conspicuously impacted, although not significantly. Considering that patients' clinical results suggest liver injury and that the liver is the site of bilirubin production during red blood cell break down, it is plausible that clinical hyperbilirubinemia and the large porphyrin pathway change (from the metabolite pathway analysis) are linked. Taken together, the significantly low erythrocyte turnover and a large shift in porphyrin metabolism in the severe group could be a potential indicator for severe COVID-19 (Liu et al., 2020b; San Juan et al., 2020). Monitoring the glycerophospholipids metabolic pathway following SARS-CoV-2 infection is of paramount importance in tracing the severity profile. Glycerophospholipids are essential components of biomembranes. These glycerophospholipids mediate signal transduction and immune activation processes in the cells. Sphingolipids regulate several processes, including growth regulation, cell migration, adhesion, apoptosis, senescence, and inflammatory responses (Matsuki et al., 2019; Ancajas et al., 2020). Thus, the differential expression of sphingolipids (see Supplementary Table 1) may have played a role in host immune and inflammatory responses to the COVID-19 infection/severity.

Significantly elevated AST, ALT, and bilirubin levels observed in our study demonstrated evidence of liver damage in the severe COVID-19 group. Liver damage affects the hepatic regulation of various lipids, including glycerophospholipids, sphingolipids, and fatty acids (Nguyen et al., 2008; Guan et al., 2020). Lipid and metabolite alterations associated with liver damage can predict COVID-19 progress and severity (Wu et al., 2020a). Host glycerophospholipid, reported to play a crucial role in the early development of enveloped viruses (Strating and van Kuppeveld, 2017; Ni et al., 2021), may be limited as the virus is actively multiplying. Most of the phospholipid markers detected in our study were from non-severe cases, which suggests that the reduced expression of glycerophospholipid molecules in severe cases could have resulted from the use of host phospholipids for SARS-CoV-2 virus multiplication. The significant activity in the glycerophospholipid metabolic pathway after SARS-CoV-2 infection is indicative that this metabolic pathway is crucial and that variations in this pathway may be applicable in tracking the progression in COVID-19 severity (Drobnik et al., 2003; Rouzer et al., 2007; Maile et al., 2018; Shen et al., 2020). Glycerophospholipid and linoleic acid metabolism pathways thus play an important role in producing metabolites that are indicative markers of the severity of SARS-CoV-2 infection. Thus, these two metabolic pathways potentially play a crucial role in producing metabolites that are usable as COVID-19 progression and severity indicators.

The lysoPE (20:3) was another notable metabolite. LysoPE (20:3) showed a significant relationship with the severity of SARS-CoV-2 infection. Glycerophospholipid metabolism may induce a possible remodeling in lipid synthesis as a result of a reduction in cholesterol and LDL (low-intensity lipoprotein), as observed in our clinical data in which it was observed that critically ill patients had lower total cholesterol levels than moderately ill COVID-19 patients. The observations echo those by Pang et al. (2021).

Since this was a descriptive observational study that used samples from two clinical trials, this study had limitations. These limitations included the low patient sample size and the study's cross-sectional and observational nature while acquiring the plasma samples. The cross-sectional acquisition of patient samples for analysis influenced the causal relationship between the identified biomarkers, and the exact internal mechanisms of metabolite changes in SARS-CoV-2 infection remain unclear. The analyzed samples were collected before the widespread detection of coronavirus variants, thus these findings do not provide any information on the new SARS-CoV-2 variants considered to have increased transmissibility, to be more virulent, and to have reduced neutralization by antibodies (monoclonal, convalescent, or post-vaccination sera) (Aleem et al., 2021; Otto et al., 2021; WHO, 2021a). There are also limitations to the role of ethnicity in the cohort, although statistical analysis revealed no significant difference in ethnicity between the groups studied. The population in the region is relatively homogeneous, with the majority being of mixed race (74.8%). The metabolomic analysis in this study was not an absolute quantification. If these findings were to be applied in clinical research and the development of diagnostic/prognostic tools, rigorous quantification and extensive validation of these molecules using standards are necessary. Finally, we acknowledge that plasma studies at different time points would have been ideal for rigorous temporal analysis. Thus, study designs involving multiple sample collecting time points need to be factored into future studies. Despite these limitations, our study presents a systemic metabolic investigation of patients that suffered non-severe and severe COVID-19 infections.

In conclusion, our study brings novel information that corroborates the importance of the metabolomic profile in patients infected with COVID-19. Incorporating clinical and laboratory data into the metabolomic analysis provides a new and indispensable perspective while seeking pragmatic COVID-19 prognostic biomarkers. We demonstrate the potential to identify metabolomic markers to predict disease progression, which may be valuable in diagnostics and individualized therapeutic interventions in managing COVID-19. These markers will also significantly increase the understanding related to the pathophysiology of COVID-19. More metabolomic studies are required since they are vital in understanding the metabolic dynamics in COVID-19, which remain unclear.

## Data Availability Statement

The original contributions presented in the study are included in the article/Supplementary Material, further inquiries can be directed to the corresponding author/s.

## Ethics Statement

This study was conducted according to principles expressed in the Helsinki Declaration. It was reviewed and approved by the local Institutional Review Board (IRB) (CEP/FMT-HVD, CAAE 4.051.475/2020). Data and samples that compose study results were derived from two clinical trials that were approved by the National Ethics Committee (CONEP) (CAAE 30152620.1.0000.0005 and CAAE 30504220.5.0000.0005). The IRB at FMT-HVD issued a waiver for obtaining informed consent forms from the participants in order to analyze the metabolomic data presented here.

## Author Contributions

GM, LO, ML, and FV: contributed to the conception and design of the study. LO, MX, FV, JD, GS, AO, and EB: data generation and collection. VM, JD, GS, AO, EB, and RC: data analysis. VM, LO, JD, MX, FV, MS, RC, FC, and GM: interpretation of results and critical appraisal. LO, VM, and JD: draft writing. FC, DB-d-S, VS, MS, FV, WM, GM, and ML: manuscript revision. All authors contributed to the article and approved the final version.

## Funding

This study was supported by *Fundação de Amparo à Pesquisa do Estado do Amazonas*- AM through the EMERGESAÚDE - AM project funds [FAPEAM EDITAL No. 005/2020 - PCTI- EMERGESAÚDE—AM] awarded to the *Fundação de Medicina Tropical Doutor Heitor Vieira Dourado*. This work also received financial support from Fundação de Amparo à Pesquisa do Estado do Amazonas (FAPEAM) through Resolution No. (s) 002/2008, 007/2018, 005/2019 and 006/2020 for MS and VS - PRO-ESTADO and POSGRAD 2021 calls; a Support Program for the Consolidation of State Education.

## Conflict of Interest

The authors declare that the research was conducted in the absence of any commercial or financial relationships that could be construed as a potential conflict of interest.

## Publisher's Note

All claims expressed in this article are solely those of the authors and do not necessarily represent those of their affiliated organizations, or those of the publisher, the editors and the reviewers. Any product that may be evaluated in this article, or claim that may be made by its manufacturer, is not guaranteed or endorsed by the publisher.

## Supplementary Material

Supplementary Figure 1 Volcano plot displaying *p*-value *vs*. log_2_FC for selecting and ranking *m/z* features for metabolite identification.

Supplementary Figure 2 Metabolites ranked based on the variable importance in projection (VIP) score.

Supplementary Figure 3 Permutation test statistics for the PLS-DA score plot. **(A)** Prediction accuracy during training; **(B)** Separation distance.

Supplementary Table 1 Characteristics of biomarkers uniquely identified from plasma samples of COVID-19 patients categorized as having severe or non-severe disease condition.
