# Supplementary figures and images for: Metabolomic Profiling of Plasma Reveals Differential Disease Severity Markers in COVID-19 Patients
Source: Front Microbiol. 2022 Apr 27;13:844283. doi: 10.3389/fmicb.2022.844283 (PMC9094083; doi:10.3389/fmicb.2022.844283)

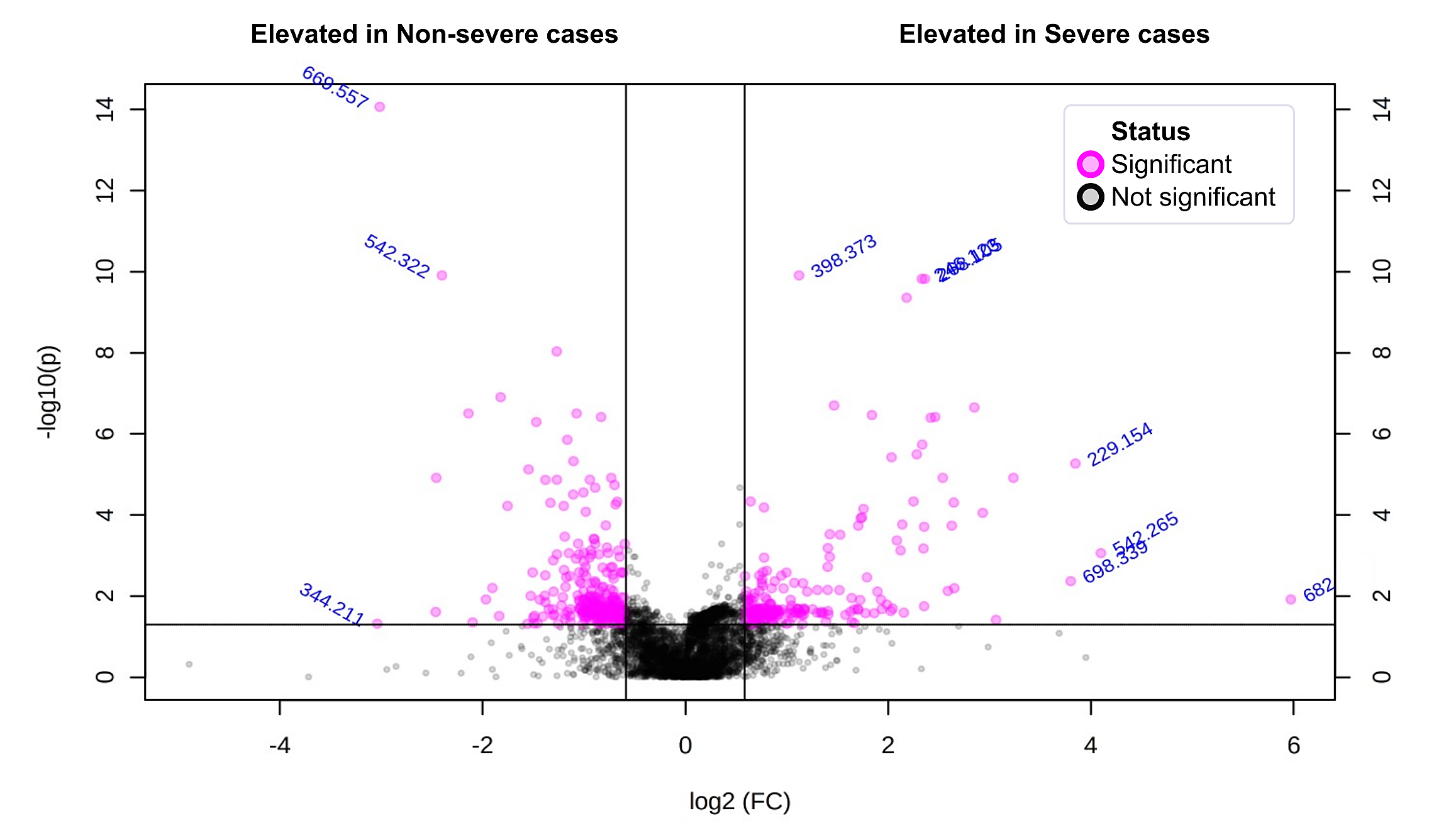

Supplement: Supplementary Figure 1 — Volcano plot displaying p-value vs. log2FC for selecting and ranking m/z features for metabolite identification. [file Image_1.tiff]

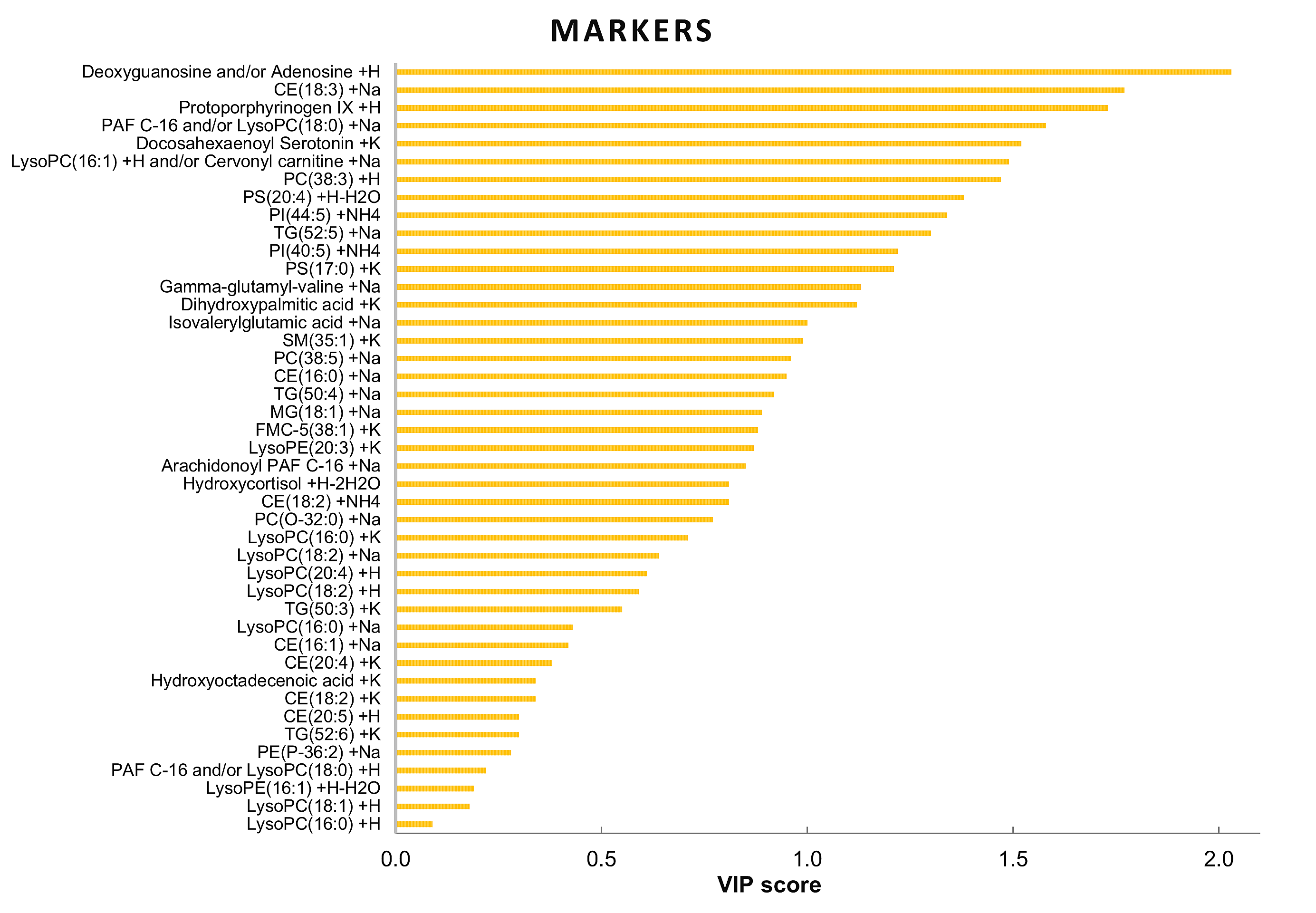

Supplement: Supplementary Figure 2 — Metabolites ranked based on the variable importance in projection (VIP) score. [file Image_2.tiff]

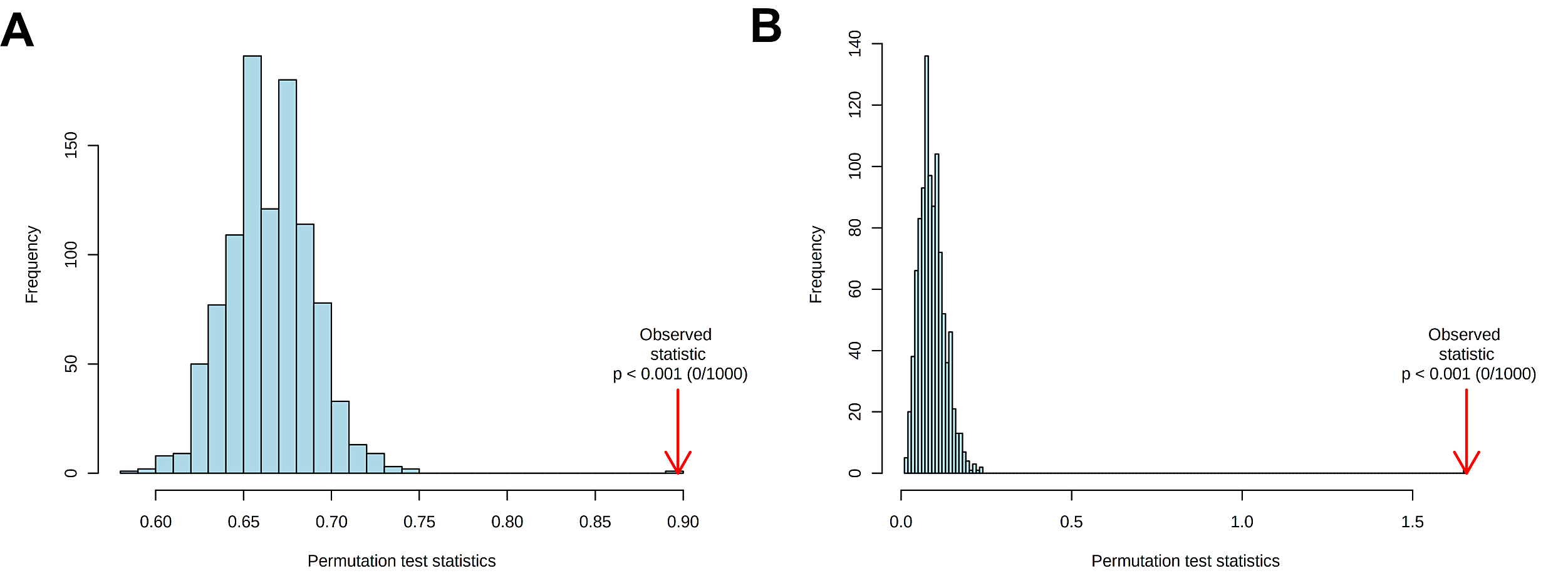

Supplement: Supplementary Figure 3 — Permutation test statistics for the PLS-DA score plot. (A) Prediction accuracy during training; (B) Separation distance. [file Image_3.tiff]
