# Supplementary material for: Metabolomic Profiling of Plasma Reveals Differential Disease Severity Markers in COVID-19 Patients
Source: Front Microbiol. 2022 Apr 27;13:844283. doi: 10.3389/fmicb.2022.844283 (PMC9094083; doi:10.3389/fmicb.2022.844283)
Supplement: Supplementary Table 1 — Characteristics of biomarkers uniquely identified from plasma samples of COVID-19 patients categorized as having severe or non-severe disease condition. [file Table_1.pdf]

Table 1 - Characteristics of biomarkers uniquely identified from plasma samples of COVID-19 patients categorized as having severe or non-severe disease condition.

Class	Molecule	Adduct	Molecular Formula	Exact *m/z*	Error (ppm)	log_2(FC)	p-value	AUC	VIP	Metlin ID	HMDB ID
**NON-SEVERE**											
Glycerolipid	TG(50:4)*	$[M+Na]^+$	$C_{53}H_{94}O_6$	849.6927	1.88	-1.38	< 0.001	0.7010	0.92	4763	HMDB0005435
	TG(50:3)*	$[M+K]^+$	$C_{53}H_{96}O_6$	867.6812	3.00	-0.64	0.021	0.6075	0.55	4751	HMDB0010417
	TG(52:5)*	$[M+Na]^+$	$C_{55}H_{96}O_6$	875.7088	0.00	-0.98	< 0.001	0.6808	1.30	4865	HMDB0005446
	TG(52:5)*	$[M+K]^+$	$C_{55}H_{96}O_6$	891.6826	1.35	-1.20	< 0.001	0.6056	0.30	4864	HMDB0005436
Phospholipid	LysoPE(16:1)*	$[M+H-H_2O]^+$	$C_{21}H_{42}NO_7P$	434.2688	-3.91	-0.77	0.014	0.5888	0.19	62290	HMDB0011504
	LysoPC(16:1)* and/or Cervonyl carnitine	$[M+H]^+$ / $[M+Na]^+$	$C_{24}H_{48}NO_7P$ / $C_{29}H_{45}NO_4$	494.3233	1.62	-1.47	< 0.001	0.6837	1.49	61693 / 58436	HMDB001038 / HMDB0006510
	LysoPC(16:0)*	$[M+H]^+$	$C_{24}H_{50}NO_7P$	496.3391	1.41	-0.67	< 0.001	0.5152	0.09	61692	HMDB0010382
	LysoPC(16:0)*	$[M+Na]^+$	$C_{24}H_{50}NO_7P$	518.3212	0.96	-0.79	< 0.001	0.7181	0.43	61692	HMDB0010382
	LysoPC(18:2)*	$[M+H]^+$	$C_{26}H_{50}NO_7P$	520.3394	0.77	-1.07	< 0.001	0.7310	0.59	61696	HMDB0010386
	LysoPC(18:1)*	$[M+H]^+$	$C_{26}H_{52}NO_7P$	522.3557	-0.57	-0.60	< 0.001	0.5811	0.18	61695	HMDB0002815
	PAF C-16 and/or LysoPC(18:0)*	$[M+H]^+$	$C_{26}H_{54}NO_7P$	524.3704	1.33	-0.69	< 0.001	0.5702	0.22	34488 / 61694	HMDB62195 / HMDB0010384
	LysoPC(16:0)*	$[M+K]^+$	$C_{24}H_{50}NO_7P$	534.295	1.12	-1.11	< 0.001	0.6404	0.71	61692	HMDB0010382
	LysoPC(18:2)*	$[M+Na]^+$	$C_{26}H_{50}NO_7P$	542.3215	0.37	-2.40	< 0.001	0.6666	0.64	61696	HMDB0010386
	LysoPC(20:4)*	$[M+H]^+$	$C_{28}H_{50}NO_7P$	544.3391	1.29	-0.66	0.001	0.6732	0.61	61705	HMDB0010395
	PAF C-16 and/or LysoPC(18:0)*	$[M+Na]^+$	$C_{26}H_{54}NO_7P$	546.3533	-0.55	-1.27	< 0.001	0.7881	1.58	34488 / 61694	HMDB006219/ HMDB0010384
	Arachidonoyl PAF C-16	$[M+Na]^+$	$C_{44}H_{82}NO_7P$	790.572	0.13	-1.00	0.001	0.6165	0.85	43414	NA
	PC(38:3)*	$[M+H]^+$	$C_{46}H_{86}NO_8P$	812.6156	0.98	-0.89	< 0.001	0.7186	1.47	39683	HMDB0008111
	PC(38:5)*	$[M+Na]^+$	$C_{46}H_{82}NO_8P$	830.5661	1.08	-1.06	0.001	0.6815	0.96	39548	HMDB0008433
	PC(O-32:0)*	$[M+Na]^+$	$C_{40}H_{82}NO_7P$	742.5713	0.95	-0.62	0.018	0.5922	0.77	40058	NA

Phospholipid - plasmalogen	PE(P-36:2)*	$[M+Na]^+$	$C_{41}H_{78}NO_7P$	750.5418	-1.33	-1.55	< 0.001	0.5803	0.28	62236	NA
Sterol	Hydroxycortisol	$[M+H-2H_2O]^+$	$C_{21}H_{30}O_6$	343.1929	-4.08	-0.63	0.024	0.5976	0.81	3170	HMDB0000418
	CE(16:1)*	$[M+Na]^+$	$C_{43}H_{74}O_2$	645.5576	0.77	-1.05	0.001	0.6252	0.42	103358	HMDB0000658
	CE(16:0)*	$[M+Na]^+$	$C_{43}H_{76}O_2$	647.5733	0.62	-0.74	0.007	0.6276	0.95	41701	HMDB0000885
	CE(18:2)*	$[M+NH_4]^+$	$C_{45}H_{76}O_2$	666.6173	1.65	-1.17	< 0.001	0.6807	0.81	41703	HMDB0000610
	CE(18:3)*	$[M+Na]^+$	$C_{45}H_{74}O_2$	669.5571	1.49	-3.02	< 0.001	0.7453	1.77	41704	HMDB0010370
	CE(20:5)*	$[M+H]^+$	$C_{47}H_{74}O_2$	671.5735	4.02	-0.87	0.018	0.5982	0.30	58532	HMDB0006731
	CE(18:2)*	$[M+K]^+$	$C_{45}H_{76}O_2$	687.5468	1.31	-0.83	< 0.001	0.6338	0.34	41703	HMDB0000610
	CE(20:4)*	$[M+K]^+$	$C_{47}H_{76}O_2$	711.5466	1.55	-0.70	< 0.001	0.6513	0.38	41709	HMDB0006726
Unsaturated Fatty Acids	Hydroxyoctadecenoic acid	$[M+K]^+$	$C_{18}H_{34}O_3$	337.2139	0.30	-0.61	0.003	0.5658	0.34	45939	HMDB0034297
**SEVERE**											
Amino acids	Isovalerylglutamic acid	$[M+Na]^+$	$C_{10}H_{17}NO_5$	254.0996	1.18	1.76	< 0.001	0.7359	1.00	5694	HMDB0000726
	Dipeptide	$[M+Na]^+$	$C_{10}H_{18}N_2O_5$	269.1094	5.2	2.03	< 0.001	0.7474	1.13	62020	HMDB0028757
Glycerolipid	MG(18:1)*	$[M+Na]^+$	$C_{21}H_{40}O_4$	379.2818	0.23	0.73	0.007	0.7399	0.89	62320	HMDB0011567
N-acyl serotonin	Docosahexaenoyl Serotonin	$[M+K]^+$	$C_{32}H_{42}N_2O_2$	525.2879	-0.19	2.09	< 0.001	0.7299	1.52	62951	NA
Phospholipid	PS(20:4)*	$[M+H-H_2O]^+$	$C_{26}H_{44}NO_9P$	528.2698	5.30	1.47	< 0.001	0.8189	1.38	46725	NA
	LysoPE(20:3)*	$[M+K]^+$	$C_{25}H_{46}NO_7P$	542.2650	-1.29	4.10	0.001	0.6632	0.87	62299	HMDB0011515
	PS(17:0)*	$[M+K]^+$	$C_{23}H_{46}NO_9P$	550.2521	3.82	2.25	< 0.001	0.6954	1.21	78857	NA
	PI(40:5)*	$[M+NH_4]^+$	$C_{49}H_{85}O_{13}P$	930.6105	-4.19	1.53	< 0.001	0.6660	1.22	80442	HMDB0009857

	PI(44:5)*	$[M+NH_4]^+$	$C_{53}H_{93}O_{13}P$	986.6740	-4.86	1.42	< 0.001	0.7384	1.34	80745	NA
Porphyrin	Protoporphyrinogen IX	$[M+H]^+$	$C_{34}H_{40}N_4O_4$	569.3137	-2.63	2.54	< 0.001	0.7172	1.73	6003	HMDB0001097
Purine	Deoxyguanosine and/or Adenosine	$[M+H]^+$	$C_{10}H_{13}N_5O_4$	268.1054	-5.22	2.36	< 0.001	0.8084	2.03	3395/86	HMDB0000085 / HMDB0000050
Sphingolipid	SM(35:1)*	$[M+K]^+$	$C_{40}H_{81}N_2O_6P$	755.5505	-5.43	0.73	0.003	0.6937	0.99	83475	HMDB0240620
	FMC-5(38:1)*	$[M+K]^+$	$C_{54}H_{95}NO_{13}$	1004.6490	-5.47	0.62	0.035	0.6532	0.88	83798	NA
Unsaturated Fatty Acids	Dihydroxypalmitic acid	$[M+K]^+$	$C_{16}H_{32}O_4$	327.1927	1.53	2.14	< 0.001	0.7311	1.12	35689	NA

* Carbon number: double bond

Markers are subjected to geometric and position isomers

Markers in red in HMDB column were not found in the system for pathway analysis

KEY: AUC (area under curve); HMDB (Human Metabolome Database); FC (Fold change);TG (Triglyceride); LysoPE (lysophosphatidylethanolamine); LysoPC (lysophosphatidylcholine); PAF C-16 (Platelet-activating factor C-16); PC (Phosphatidylcholine); PE (Phosphatidylethanolamine); CE (Cholesteryl ester); MG (Monoacylglycerol); PS (Phosphatidylserine); PI (Phosphotidylinositol); SM (Sphingomyelin); FMC (fast-migrating cerebrosides); VIP (variable importance in projection); NA (Not Applicable)
